# TISMO: syngeneic mouse tumor database to model tumor immunity and immunotherapy response

**DOI:** 10.1093/nar/gkab804

**Published:** 2021-09-17

**Authors:** Zexian Zeng, Cheryl J Wong, Lin Yang, Nofal Ouardaoui, Dian Li, Wubing Zhang, Shengqing Gu, Yi Zhang, Yang Liu, Xiaoqing Wang, Jingxin Fu, Liye Zhou, Boning Zhang, Sarah Kim, Kathleen B Yates, Myles Brown, Gordon J Freeman, Ravindra Uppaluri, Robert Manguso, X Shirley Liu

**Affiliations:** Department of Data Science, Dana Farber Cancer Institute, Boston, MA 02215, USA; Department of Biostatistics, Harvard T.H. Chan School of Public Health, Boston, MA 02215, USA; Department of Data Science, Dana Farber Cancer Institute, Boston, MA 02215, USA; Department of Biomedical Informatics, Harvard Medical School, Boston, MA 02115 USA; Department of Data Science, Dana Farber Cancer Institute, Boston, MA 02215, USA; Department of Data Science, Dana Farber Cancer Institute, Boston, MA 02215, USA; Department of Data Science, Dana Farber Cancer Institute, Boston, MA 02215, USA; Department of Data Science, Dana Farber Cancer Institute, Boston, MA 02215, USA; School of Life Science and Technology, Tongji University, Shanghai, 200060, China; Department of Medical Oncology, Dana-Farber Cancer Institute, Boston, MA 02215, USA; Center for Functional Cancer Epigenetics, Dana-Farber Cancer Institute, Boston, MA 02215, USA; Department of Data Science, Dana Farber Cancer Institute, Boston, MA 02215, USA; Department of Biostatistics, Harvard T.H. Chan School of Public Health, Boston, MA 02215, USA; Department of Data Science, Dana Farber Cancer Institute, Boston, MA 02215, USA; Department of Biostatistics, Harvard T.H. Chan School of Public Health, Boston, MA 02215, USA; Department of Data Science, Dana Farber Cancer Institute, Boston, MA 02215, USA; Department of Medical Oncology, Dana-Farber Cancer Institute, Boston, MA 02215, USA; Center for Functional Cancer Epigenetics, Dana-Farber Cancer Institute, Boston, MA 02215, USA; Department of Data Science, Dana Farber Cancer Institute, Boston, MA 02215, USA; Department of Biostatistics, Harvard T.H. Chan School of Public Health, Boston, MA 02215, USA; Department of Medical Oncology, Dana-Farber Cancer Institute, Boston, MA 02215, USA; Broad Institute of Harvard and Massachusetts Institute of Technology, Cambridge, MA 02129, USA; Department of Medical Oncology, Dana-Farber Cancer Institute, Boston, MA 02215, USA; Department of Medical Oncology, Dana-Farber Cancer Institute, Boston, MA 02215, USA; Center for Functional Cancer Epigenetics, Dana-Farber Cancer Institute, Boston, MA 02215, USA; Department of Data Science, Dana Farber Cancer Institute, Boston, MA 02215, USA; Department of Biostatistics, Harvard T.H. Chan School of Public Health, Boston, MA 02215, USA; Broad Institute of Harvard and Massachusetts Institute of Technology, Cambridge, MA 02129, USA; Broad Institute of Harvard and Massachusetts Institute of Technology, Cambridge, MA 02129, USA; Center for Cancer Research, Massachusetts General Hospital, Harvard Medical School, Charlestown, MA 02114, USA; Department of Medical Oncology, Dana-Farber Cancer Institute, Boston, MA 02215, USA; Center for Functional Cancer Epigenetics, Dana-Farber Cancer Institute, Boston, MA 02215, USA; Department of Medical Oncology, Dana-Farber Cancer Institute, Boston, MA 02215, USA; Department of Medical Oncology, Dana-Farber Cancer Institute, Boston, MA 02215, USA; Department of Surgery, Brigham and Women's Hospital, Boston, MA 02215, USA; Broad Institute of Harvard and Massachusetts Institute of Technology, Cambridge, MA 02129, USA; Center for Cancer Research, Massachusetts General Hospital, Harvard Medical School, Charlestown, MA 02114, USA; Department of Data Science, Dana Farber Cancer Institute, Boston, MA 02215, USA; Department of Biostatistics, Harvard T.H. Chan School of Public Health, Boston, MA 02215, USA; Center for Functional Cancer Epigenetics, Dana-Farber Cancer Institute, Boston, MA 02215, USA

## Abstract

Syngeneic mouse models are tumors derived from murine cancer cells engrafted on genetically identical mouse strains. They are widely used tools for studying tumor immunity and immunotherapy response in the context of a fully functional murine immune system. Large volumes of syngeneic mouse tumor expression profiles under different immunotherapy treatments have been generated, although a lack of systematic collection and analysis makes data reuse challenging. We present Tumor Immune Syngeneic MOuse (TISMO), a database with an extensive collection of syngeneic mouse model profiles with interactive visualization features. TISMO contains 605 *in vitro* RNA-seq samples from 49 syngeneic cancer cell lines across 23 cancer types, of which 195 underwent cytokine treatment. TISMO also includes 1518 *in vivo* RNA-seq samples from 68 syngeneic mouse tumor models across 19 cancer types, of which 832 were from immune checkpoint blockade (ICB) studies. We manually annotated the sample metadata, such as cell line, mouse strain, transplantation site, treatment, and response status, and uniformly processed and quality-controlled the RNA-seq data. Besides data download, TISMO provides interactive web interfaces to investigate whether specific gene expression, pathway enrichment, or immune infiltration level is associated with differential immunotherapy response. TISMO is available at http://tismo.cistrome.org.

## INTRODUCTION

Immunotherapies targeting co-inhibitory pathways have shown remarkable clinical success but only demonstrate efficacy in a subset of cancer patients (1). The underlying mechanisms of heterogeneous response to immune checkpoint blockade (ICB) therapy remain unclear. Clinical samples from patient tumors accurately represent the tumor microenvironment (TME), but are difficult to access and conduct controlled experiments. Pre-clinical models that faithfully recapitulate the complexity of cancer cells and their interactions with the immune system are essential for investigating potential resistance mechanisms to ICB (2). Commonly used *in vitro* systems for cancer research, such as conventional 2D cell culture or 3D organoids, are inadequate to model the complexity of the TME. Instead, syngeneic tumors transplanted into immunocompetent mice are readily available and provide reproducible results for cancer immunology research. Syngeneic mouse models have been widely used in cancer immunology studies, and a large volume of tumor expression profiles under various immunotherapy treatments have been generated (3,4). However, lack of systematic collection and variation in analysis of individually published syngeneic tumor profiles makes data reuse challenging.

Multiple existing data resources are available for mouse models of cancer, such as MPD (5,6), MMHCdb (7,8), MGD (9), GXD (10), PDX Finder (11) and NCI OMF (https://oncologymodels.org/annotatedDataSets). Among these databases, only NCI OMF contains syngeneic tumor model studies, although it solely provides meta-information of these studies without expression profiles. Similar to NCI OMF in providing study-level meta-information, GXD (10) focuses on expression profiles of wild-type and genetic mutant mice, but its scope is limited to embryonic stages and postnatal period. MPD (5,6) focuses on phenotypes of different mouse strains under specific experimental treatments, with strain-specific genotyping and microarray gene expression data for selected samples, although does not include syngeneic tumor models. MMHCdb (7,8) focuses on genetically engineered mouse models, inbred strains, and patient-derived xenograft models of human cancer and provides information about specific mutations/allelic variants in mouse tumors. MGD (9) is a major component of Mouse Genome Informatics (MGI) and provides descriptive annotations about mouse genes and other genome features such as nucleotide, protein sequences, and SNPs. Both MMHCdb (7,8) and MGD (9) explore the association between human diseases and mouse models by linking genetic background to phenotype, but neither include syngeneic tumor models. PDX Finder (11) is a searchable catalogue containing information for 1985 PDX models of diverse cancers, but as the name implies, this resource is limited to PDX models. To the best of our knowledge, there is no published database with a comprehensive collection of syngeneic mouse tumors that provides expression profiles and phenotypic data.

Herein, we present Tumor Immune Syngeneic MOuse (TISMO), a large-scale publicly accessible resource of syngeneic mouse models. TISMO (http://tismo.cistrome.org) is a comprehensive database with over two thousand uniformly processed and quality-controlled RNA-seq samples of syngeneic mouse cancer cell lines and tumor models. These datasets were uniformly processed from raw sequencing reads using a standardized workflow. In addition, immune cell infiltration and pathway enrichment levels have been inferred and phenotypic metadata have been manually annotated. TISMO provides interactive web interfaces for users to compare and visualize gene expression, pathway enrichment, and immune infiltration level across syngeneic mouse models, treatments, and response groups. The continued maintenance of TISMO will be of great utility to the cancer immunology and immuno-oncology research community.

## MATERIALS AND METHODS

### Data collection and meta information curation

We developed a parser to query datasets deposited in the Gene Expression Omnibus (GEO) (12) between 2016 and 2021. Using this parser, we performed keyword searches to identify studies matching a list of manually curated syngeneic mouse models or syngeneic cancer cell lines (Supplementary Table S1). For matched studies, meta-files containing study design and sample information were downloaded through the parser. We manually curated and confirmed each sample for database inclusion. We also annotated syngeneic mouse model phenotypes through literature searches. In total, we collected 1868 syngeneic tumor or cell line RNA-seq samples from 137 published studies. We also included 255 in-house RNA-seq samples generated by ourselves which have not been published before.

### Transcriptome data processing

To ensure consistency, we downloaded raw sequencing reads from each study and processed the data through a standardized pipeline called RNA-seq IMmune Analysis Pipeline (RIMA, https://liulab-dfci.github.io/RIMA). RIMA is an automated Snakemake pipeline developed by our group to streamline the processing of RNA-seq data, including but not limited to read alignment, quality control, expression qualification, batch effect removal, and immune cell infiltration inference. FASTQ files containing the raw reads were downloaded or transferred. Read alignments were performed with STAR (13) (v.2.4.2a) against the mm10 reference genome assembly (mm10, Genome Reference Consortium Mouse Build 38) from the NCI Genomic Data Commons (GDC). RNA-seq quality control (QC) was performed on the aligned BAM files using RSeQC (14) (v2.4). With the reads appropriately aligned, expression levels were quantified by SALMON (15) (v.0.14.0) on the BAM files. Ensemble IDs were converted to mouse gene symbols (GRCm38.p6).

We also characterized pathway enrichment for each sample to enable comparison between conditions. From Molecular Signatures Database (MSigDB) (16), we first collected 17456 gene-sets, including 7479 GO biological processes, 996 GO cellular components, 1704 GO molecular functions (17), 186 Kyoto Encyclopedia of Genes and Genomes (KEGG) pathways (18), 4872 immunologic signatures (16), 615 wiki pathways, and 1604 Reactome pathways (19). Then for each RNA-seq profile, we evaluated the level of each pathway by single sample gene set enrichment analysis (ssGSEA) (20). For user-defined gene set with weights, we calculate the weighted sum expression of the gene set following the equation of $p\; = \mathop \sum \limits_{i = 1}^n {k_i}{\rm{log}}( {{e_i}} )\;$, where $p$ denotes the pathway level, ${k_i}$ denotes the weights, and ${e_i}$ denotes the transcripts per kilobase million (TPM) of the *i*th gene in the pathway.

Samples collected from different studies were processed with different protocols and platforms, and subject to technical bias. Even though TISMO conducts all sample comparisons within each cohort, we normalized the data for consistency across cohorts. Specifically, for each cohort we pre-computed false discovery rates (FDR) using DESeq2 (21) for each gene and comparison condition. To aid visualization, we normalized the transcriptome TPM data by quantile normalization to calibrate the scaling and distribution differences across samples, separately in each syngeneic model. We then performed batch effect correction between studies using ComBat within each syngeneic model (22). Notably, the combination of quantile normalization and ComBat was reported to achieve the best performance for batch effect removal in a recent benchmark study (23). In an evaluation, we observed that after quantile normalization and batch effect removal, the samples’ housekeeping-gene-signatures, averaged by 600 housekeeping genes (24), are less scattered between samples from different studies (Supplementary Figure S1A–D). The coefficient of variations reduced from 0.12 to 0.06 and from 0.14 to 0.09 for the *in vitro* samples and *in vivo* samples, respectively.

To make reliable and robust immune cell infiltration estimations, we utilized Immunedeconv (25), an R package that integrates state-of-the-art algorithms for immune deconvolution, including TIMER (26), xCell (27), CIBERSORT (28), EPIC (29), quanTIseq (30). We also incorporated a murine-specific immune infiltration deconvolution tool, mMCPcounter (31). Although each algorithm has unique properties and strengths (25), immune infiltration estimations supported by multiple algorithms provide more confident results. The source code to search mouse-related studies, download sequencing data, perform batch effect correction and infer immune infiltration were deposited at the Github repository (https://github.com/zexian/TISMO_data).

### Website development

To enable users to systematically explore the curated datasets, we developed a user-friendly web interface to host the TISMO database. The TISMO website is freely available at http://tismo.cistrome.org without any registration or login restriction. It is implemented with the R-Shiny framework (R version 3.6.3) on an Apache2 HTTP server, and is compatible with smartphones and tablets. The website consists of seven functional components: ‘Home’, ‘Data Browser’, ‘Gene’, ‘Pathway’, ‘Infiltrates’, ‘Data Download’, and ‘Documentation’. ‘Home’ includes a tutorial video with step-by-step instructions on using the database and website. Users could browse or search the curated metadata using the ‘Data Browser’ module to locate relevant syngeneic models. ‘Gene’, ‘Pathway’ and ‘Infiltrates’ modules enable users to select and compare gene expression, pathway enrichment, and immune infiltration level between treatments, response groups, and models. Users could explore and derive gene expression programs or immune infiltrates consistently associated with ICB treatment and response in these modules. In the pathway module, users could also upload and evaluate self-defined gene sets. In the ‘Data Download’ module, users could download phenotypic metadata, quantified gene expression, and immune cell infiltration for all samples in the database. The documentation page summarizes the data processing steps and the number of samples in different models and treatment conditions.

## RESULTS

### Data summary

**Figure 1.**
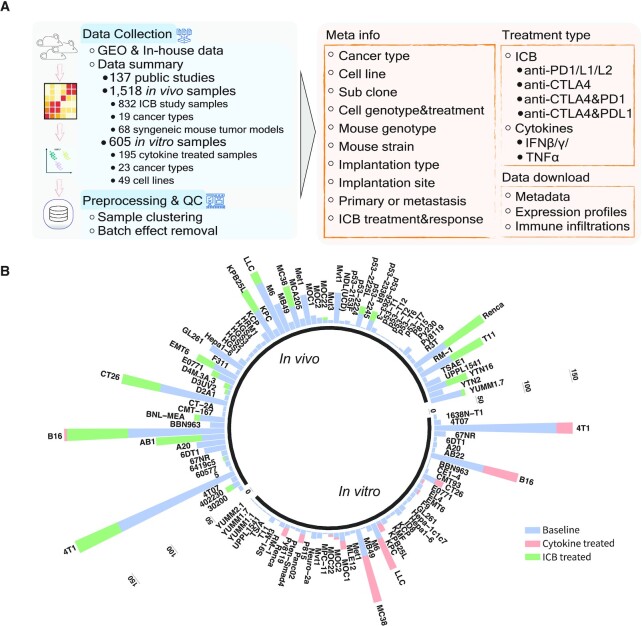
Overview of the TISMO database and summary of data available for download. (**A**) The datasets hosted at the TISMO database were curated from public domains and in-house data. In total, TISMO contains 605 *in vitro* samples, of which 195 were cytokine treated. TISMO also contains 1518 *in vivo* samples, of which 832 samples were from immune checkpoint blockade (ICB) studies. All samples were uniformly processed from raw sequencing reads within a standardized workflow, including alignment, quality control, expression qualification, batch effect removal, and immune cell infiltration inference. In addition, sample metadata, including cell line, mouse strain, transplantation site, ICB treatment, survival and response status were manually annotated from publications. The metadata, expression profiles, and immune cell expression profiles for all samples can be downloaded from the TISMO database. (**B**) TISMO contains *in vitro* RNA-seq samples from 49 syngeneic cancer cell lines across 23 cancer types. TISMO also contains *in vivo* samples from 68 syngeneic mouse tumor models across 19 cancer types. Samples treated with cytokines (IFNγ, IFNβ, TNFα) are labeled in pink; samples treated with ICB (anti-PD1, anti-PDL1, anti-PDL2, antiCTLA4 or their combinations) are labelled in green.

The current TISMO database includes 605 *in vitro* RNA-seq samples from 49 syngeneic cancer cell lines across 23 cancer types. TISMO also contains 1518 *in vivo* RNA-seq samples from 68 syngeneic mouse tumor models across 19 cancer types (Figure 1A and B). Many samples within the TISMO database have undergone different treatments, including anti-PD1, anti-PDL1, anti-PDL2, anti-CTLA4, interferon γ (IFNγ), IFNβ, tumor necrosis factor alpha (TNFα), or in combination with other treatments (Supplementary Tables S2 and S3). We manually annotated phenotypic data for each sample by referencing the original article, including cancer type, cancer cell line, cell treatment, cell genotype, mouse genotype, mouse strain, implantation type, implantation site, mouse ICB treatment, and response status (Supplementary Tables S2-S3). In addition, we have collected available survival information from published studies (Supplementary Figure S2). Gene expression levels were quantified for all samples, and immune cell infiltration levels for each *in vivo* sample were inferred based on expression profiles (Supplementary Table S4). All curated metadata, expression data, and immune infiltration estimation data can be downloaded from the ‘Data Download’ module in the TISMO database.

### Exploration of gene expression and pathway enrichment changes induced by cytokine treatment

**Figure 2.**
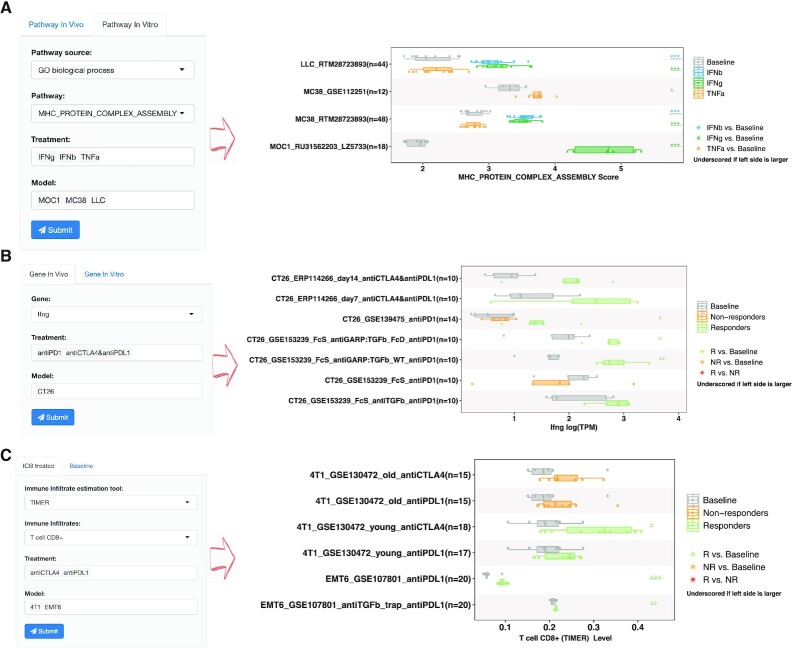
Examples of TISMO features exploring factors associated with cytokine treatment and immunotherapy response. (**A**) Users could select pathways, cytokines, and syngeneic cell lines to evaluate their expressions before and after cytokine treatment. (B, C) Users could select a gene (**B**) and immune cell infiltration (**C**) to evaluate whether its level changes upon ICB treatment, and compare it between ICB responders and non-responders. Immune cell infiltration levels were inferred by six different state-of-the-art deconvolution algorithms. The generated figures could be downloaded in jpg, pdf, and table formats.

Cytokines, including IFN and TNF, play essential roles in adaptive immunity in the TME (32–35). Due to their critical roles in anti-tumor immunity, it is of great interest to know how gene expression or pathway enrichment is differentially regulated by IFN or TNF stimulation. In TISMO, the *in vitro* data allow users to explore the effects of cytokine treatments on syngeneic cancer cell lines. After a user selects a gene or gene set, cytokine, and cell line, the TISMO webserver displays box plots of gene expression or pathway enrichment before and after cytokine treatment. For example, Figure 2A demonstrates how a user could investigate the ‘MHC_PROTEIN_COMPLEX_ASSEMBLY’ pathway after cytokine stimulation in syngeneic cancer cell lines. After selecting the pathways, cytokines, and cell lines of interest, and submitting the query, a summary boxplot of the pathway level before and after cytokine treatment is generated together with statistical comparisons. As expected, there is a significant increase in major histocompatibility complex (MHC) protein complex after IFNγ and IFNβ stimulation (32,36). In addition to the curated pathways, users could compare gene expression in the ‘Gene’ module or upload their own gene set of interest in the ‘Upload pathway’ module. The differentially expressed genes and pathway enrichment between comparison groups are statistically evaluated by the Wald test using DESeq2 (21) and the Student's t-test, respectively. In TISMO, we have curated 17456 pathways from MSigDB (16) and have characterized pathway enrichment for each sample using ssGSEA (20), which users can explore interactively through the web interface. Users could also upload self-defined gene sets with the flexibility to adjust these gene weights. If a user uploads a customized gene set, TISMO will calculate its level in each *in vitro* and *in vivo* sample (Materials and Methods), allowing users to evaluate them across models, treatments, and response groups.

### Comparison of gene expression, pathway enrichment, and immune infiltration between ICB treatment and response groups

ICB treatments, including anti-CTLA4 and anti-PD1/L1 provide clinical benefits in only a subset of patients (37). Mechanisms underlying heterogeneous ICB response remain an open question and the association between immune infiltration and ICB response is of great interest to many researchers (26). In addition to gene expression profiles and pathway enrichment, the TISMO database integrates inferred immune infiltrations from six state-of-the-art immune cell deconvolution algorithms, including TIMER (26), xCell (27), CIBERSORT (28), EPIC (29), quanTIseq (30) and mMCPcounter (31). We have annotated treatment and response information for ICB study samples (*N* = 832), allowing users to explore whether specific gene expression programs or immune infiltrations are robustly associated with ICB response or resistance. Using TISMO, users could select a specific gene, pathway, or immune cell infiltration, evaluate whether its level changes upon ICB treatment, and compare it between ICB responders and non-responders (Figure 2B, C). For example, Figure 2B shows how the IFNγ gene expression in CT26 model is stimulated by different ICB treatments. After a user selects genes, ICB treatments, and syngeneic mouse models of interest, TISMO website will generate a box plot summarizing the gene expression levels before and after ICB treatments in different comparison conditions. The expression levels are grouped by syngeneic model, treatment group, and response status in the figure. After ICB treatment, the IFNγ expression levels in CT26 model are significantly upregulated in the ICB responders, but not in the non-responders (Figure 2B) (32). In addition to gene expression levels, users could also compare pathway enrichment levels in the ‘Pathway’ module or immune cell infiltration in the ‘Infiltrate’ module (Figure 2C). The differential gene expression between groups is statistically evaluated by the Wald test in DESeq2 (21), and the differential pathway enrichment (characterized by ssGSEA) (20)) and immune infiltration are evaluated by the Mann–Whitney *U* test. Compared to clinical samples, syngeneic tumor models allow scientists to evaluate immunotherapy response in a more controlled and reproducible manner. TISMO’s website enables users to efficiently evaluate genes, pathways, and immune cell infiltration in the context of ICB treatment, to generate or validate hypotheses on immunotherapy response.

### Exploration of meta information, figure generation and data download

TISMO’s website hosts a data browser module to help users locate relevant mouse models from our collection. Users could query sample Study ID (the majority representing the GSE ID, a study identification number for the GEO database), sample metadata, treatment condition, response status and the number of replicates in each design. The data browser module aids researchers in selecting the most relevant syngeneic mouse models to supply evidence for hypothesis generation or validation. On the TISMO website, data cohorts and treatment types could be selected for statistical comparison and visualization. The interactive filtering and visualization features enable users to systematically compare different models, cell lines, treatments, and response groups. The generated figures could be downloaded in jpg, pdf, and table formats. The download page provides access to the expression matrices of all 2123 RNA-seq profiles, immune cell infiltration estimations of the *in vivo* samples, and the manually annotated metadata for all samples.

## DISCUSSION

Syngeneic mouse models are essential in immunotherapy research as they enable the study of cancer cells in the context of immunocompetent hosts. Large amounts of syngeneic mouse model profiles have been generated, but these data are scattered, making data reuse challenging. There is still no comprehensive, intuitive, and convenient database with user-friendly, interactive web features for researchers to explore syngeneic mouse model data. TISMO (http://tismo.cistrome.org) is the first comprehensive database for users to investigate and visualize gene expression, pathway enrichment, and immune cell infiltration levels in syngeneic mouse models across different ICB treatment and response groups. Expression profiles hosted on TISMO were uniformly processed from the raw sequencing reads. The immune infiltration levels were inferred by six state-of-the-art deconvolution algorithms, and the metadata were manually annotated from publications. TISMO provides web interfaces to help users explore the syngeneic mouse model data interactively. In summary, TISMO is a comprehensive database of syngeneic mouse models that will help users select relevant syngeneic mouse tumor models, provide data to generate and test hypotheses, and reveal novel mechanisms of ICB response and resistance.

## DATA AVAILABILITY

TISMO is available at http://tismo.cistrome.org to all users without restrictions. Annotated mouse syngeneic metadata are available on the website. All expression data and immune infiltration estimation can be downloaded from the data download page.

## Supplementary Material

gkab804_Supplemental_Files
