# Supplementary material for: TISMO: syngeneic mouse tumor database to model tumor immunity and immunotherapy response
Source: Nucleic Acids Res. 2021 Sep 17;50(D1):D1391–7. doi: 10.1093/nar/gkab804 (PMC8728303; doi:10.1093/nar/gkab804)
Supplement: gkab804_Supplemental_Files [file gkab804_supplemental_files.zip › TISMO_supplementary_file-.pdf]

TISMO: syngeneic mouse tumor database to model tumor immunity and immunotherapy response

Supplementary information

Figure S1. Housekeeping gene signature level before and after quantile normalization and batch effect correction. The signature level is the average across 600 housekeeping genes **(A)***in vitro* samples before quantile normalization and batch effect correction. **(B)** *in vivo* samples before quantile normalization and batch effect correction. **(C)** *in vitro* samples after quantile normalization and batch effect correction. **(D)** *in vivo* samples after quantile normalization and batch effect correction.

Figure S2. Tumor volume and mouse survival data collected for the ICB related studies byreferencing the original articles.
